# Surgeon–Pathologist Team Approach Dramatically Affects Lymph Nodes Detection and Improves Patients’ Short-Term Outcome

**DOI:** 10.3390/cancers14041034

**Published:** 2022-02-18

**Authors:** Maria Raffaella Ambrosio, Bruno Perotti, Alda Battini, Caterina Fattorini, Andrea Cavazzana, Rocco Pasqua, Piergaspare Palumbo, Liano Gia, Marco Arganini

**Affiliations:** 1 Pathology Unit, Azienda Sanitaria Toscana Nord-Ovest, Via Cocchi 1, 56121 Pisa, Italy; caterina.fattorini@uslnordovest.toscana.it (C.F.); andrea.cavazzana@libero.it (A.C.); 2 Surgery Unit, Ospedale Unico Versilia and Nuovo Ospedale Apuane, Azienda Sanitaria Toscana Nord Ovest, 56121 Pisa, Italy; bruno.perotti@uslnordovest.toscana.it (B.P.); alda.battini@ulsnordovest.toscana.it (A.B.); liano.gia@uslnordovest.toscana.it (L.G.); marco.arganini@uslnordovest.toscana.it (M.A.); 3 Department of Surgical Sciencies, University “La Sapienza”, 00100 Roma, Italy; rocco.pasqua@live.it (R.P.); piergaspare.palumbo@uniroma1.it (P.P.)

**Keywords:** gastric cancer, lymph node harvesting, downstaging, skip metastasis

## Abstract

**Simple Summary:**

Appropriate lymph node harvesting for patients with gastric cancer is fundamental for a correct staging and is strongly related to survival. In this study, we present a new protocol for on-site macroscopic evaluation and sampling of lymph nodes for gastric cancer patients. With the joint collaboration of surgeons and pathologists, our method aims to provide the largest possible number of analyzed lymph nodes per patient, allowing for a better staging. We are convinced that this approach is routinely feasible, and our preliminary results seem to confirm better patient stratification compared to other lymph node dissection methods.

**Abstract:**

The downstaging of gastric cancer has recently gained particular attention in the field of gastric cancer surgery. The phenomenon is mainly due to an inappropriate sampling of lymph nodes during standard lymphadenectomy. Hence, collection of the maximum number of lymph nodes is a critical factor affecting the outcome of patients. None of the techniques proposed so far have demonstrated a real efficiency in increasing the number of identified lymph nodes. To harvest the maximum number of lymph nodes, we designed a protocol for on-site macroscopic evaluation and sampling of lymph nodes according to the Japanese Gastric Cancer Association protocol. The procedure was carried out by a surgeon/pathologist team in the operating room. We enrolled one hundred patients, 50 of whom belonged to the study group and 50 to a control group. The study group included patients who underwent lymph node dissection following the proposed protocol; the control group encompassed patients undergoing standard procedures for sampling. We compared the number and maximum diameter of lymph nodes collected in both groups, as well as some postoperative variables, the 30-day mortality and the overall survival. In the study group, the mean number of lymph nodes harvested was higher than the control one (*p* = 0.001). Moreover, by applying the proposed technique, we sampled lymph nodes with a very small diameter, some of which were metastatic. Noticeably, no difference in terms of postoperative course was identified between the two groups, again supporting the feasibility of an extended lymphadenectomy. By comparing the prognosis of patients, a better overall survival (*p* = 0.03) was detected in the study group; however, to date, no long-term follow-up is available. Interestingly, patients with metastasis in node stations number 8, 9, 11 or with skip metastasis, experienced a worse outcome and died. Based on our preliminary results, the pathologist/surgeon team approach seems to be a reliable option, despite of a slight increase in sfaff workload and technical cost. It allows for the harvesting of a larger number of lymph nodes and improves the outcome of the patients thanks to more precise staging and therapy. Nevertheless, since a higher number of patients are necessary to confirm our findings and assess the impact of this technique on oncological outcome, our study could serve as a proof-of-concept for a larger, multicentric collaboration.

## 1. Introduction

Gastric cancer (GC) is the fourth most common malignancy worldwide; although its incidence is declining in the Western world, this disease still remains the second leading cause of cancer related death in both sexes [1]. The Japanese Gastric Cancer Association (JGCA) has recently defined the treatment guidelines of GC according to the tumor stage [2], precisely stratifying lymph node (LN) dissection according to the wideness of lymphatic resection [3]. As LN metastases occur early during the progression of the disease, JGCA highlighted the importance of collecting the higher number of epi- and peri-gastric detectable LNs [3]. Accordingly, curative gastrectomy (with no macroscopic or microscopic residual tumor tissue) plus D2 LN dissection has been regarded as the standard surgery for potentially curable T2-4 tumors as well as cT1 N-positive tumors [4], with demonstrated decreased regional recurrence and improved long-term survival for patients [5]. Both the eighth edition of the TNM [6] and the last American Joint Committee on Cancer (AJCC) Staging System [7] recommend a minimum number of 16 LNs to ensure reliable node (N) staging. A retrieved LNs count of less than 16 LNs accounts for downstaging, which usually occurs in 10–25% of patients, and affects treatment plans and prognosis [8,9,10,11,12]. In fact, inadequate LN dissection can lead to residual cancer cells spreading to LNs, in the form of isolated tumor cells, micrometastasis (maximum size of 2 mm) or solitary single-LN metastasis (SLM) [3]. This results in a higher recurrence rate after surgery [5,13]. At least 5% to 15% of SLM are skip metastases which are metastases bypassing the normal lymphatic stream, not related to primary tumor site [i.e., lower-third tumor metastatic to number (no.) 7 station]. SLM usually occurs along the left gastric artery (station no. 7), or involves the central LN (CnLN) compartment. CnLN compartment covers the anterior common hepatic artery (station no. 8), the coeliac trunk (station no. 9) and the splenic artery (station no. 11p) [14]. Many theories have been proposed to explain such an occurrence, most of them dealing with the complex, multidirectional nature of the lymphatic drain of the stomach [3,13,15]. It has been demonstrated that patients showing skip metastasis or multiple metastatic stations, especially in the CnLN compartment, experience shorter survival [16]. Only a higher LNs removal may allow for identifying skip metastasis and SLM. Therefore, the AJCC strongly encourages sampling and assessment of over 30 LNs, leading to the conclusion that the number of LNs dissected as well as the total number of positive-to-negative LNs (i.e., LN ratio—LNR) represent independent prognostic factors [9]. Both parameters are helpful to enhance the rate of curative resection and reduce the incidence of local recurrence, finally improving the overall survival (OS) rate [8,9,10].

The mean number of harvested LNs in D2 lymphadenectomy varies between 25 and 52 in different published series [17,18]. Inadequate LNs harvesting may depend either on the surgeon’s technical skill and the pathologist’s experience or on both. In fact, the surgeon could remove few LNs, and similarly the pathologist may achieve a suboptimal LN retrieval from formalin-fixed (FF) specimens. In Japan and in specialized Western Centers, the surgeon himself harvests LNs on fresh tissue in the operating room (OR), whereas in nonspecialized Western Institutions, LNs sampling is carried out by the pathologist on FF specimens in the grossing room (GR) [19].

To overcome the standoff situation of two comprehensive but seemingly concurrent approaches, we proposed and standardized a procedure of LNs on-site evaluation involving both surgeon and pathologist in the OR. The primary endpoint of the study was to evaluate if such an approach increases the number of detected LNs, providing a precise LNR. The secondary endpoint regarded the impact of D2 lymphadenectomy on mortality, morbidity and OS of GC patients. Moreover, we aimed to demonstrate if our approach is feasible and routinely applicable and could hopefully set a frame for future studies and discussion.

## 2. Materials and Methods

### 2.1. Methods

The study was carried out by means of a prospective phase and a retrospective review of a prospective maintained database for GC at Azienda Sanitaria Toscana Nord-Ovest, based on a pivotal communication by our group [20]. This study was conducted according to the STROBE Guidelines and under the permission of the Ethic Committee of Azienda Sanitaria Toscana Nord Ovest. The need for informed written consent was waived due to the retrospective nature of the study.

**Figure 1:**
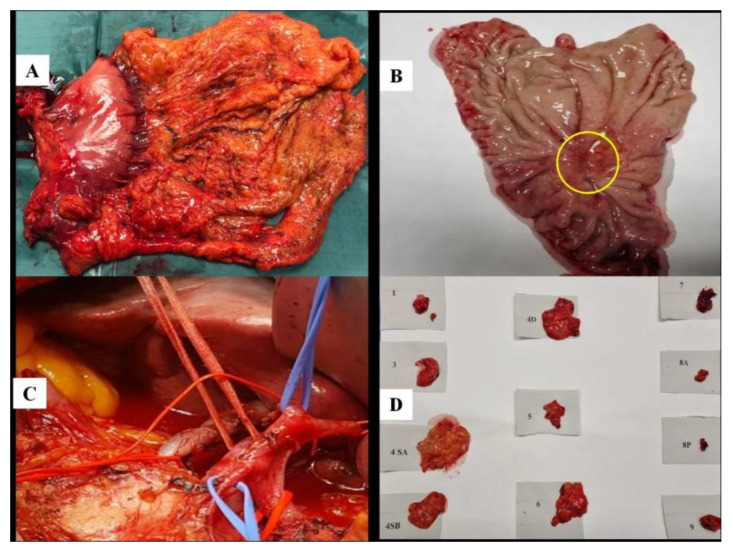
Lymph node harvesting according to the Japanese Gastric Cancer Protocol. (**A**): en bloc resection of stomach, omentum, lesser and greater curve adipose tissue. (**B**): the gastric specimen after perigastric lymph node harvesting, showing an ulcerated lesion of the angulus (encircled). (**C**): the surgical bed post central compartment lymph nodes removal. (**D**): the different lymph node stations separated and picked up.

The prospective phase started soon after the initial wave of the SARS-CoV-2 pandemic. We enrolled the first 50 patients presenting to the Surgery Unit of Ospedale Unico della Versilia (Lido di Camaiore; Lucca, Italy) and Nuovo Ospedale Apuane (Massa, Italy) with a diagnosis of GC according to the latest World Health Organization (WHO) classification [21]. Exclusion criteria included esophagogastric junction and stump tumors, squamous cell and neuroendocrine histotype, and stromal histogenesis. All patients underwent a multidisciplinary evaluation followed by a staging process including tumor marker analysis and total body computed tomography (CT) scanning. They were all submitted to a subtotal (either distal and proximal)/total gastric resection with curative intent [2]. As stated in the last “Associazione Italiana di Oncologia Medica” guidelines [2,22], patients affected by an early form of GC underwent a D1 LN dissection; in the others, a D2 lymphadenectomy was applied according to the JGCA protocol [2]. For reconstruction, the Roux-ex-Y technique was performed in all cases. After total gastrectomy, esophagojejunostomy using an EEA stapler (diameter 25 mm) was used routinely. In subtotal gastrectomy, Roux-en-Y gastrojejunostomy was accomplished using a linear stapler (60 mm). All procedures were standardized and carried out in turn by two surgeons and two pathologists skilled in gastrointestinal oncology. The sampling was completed in the OR by on-site evaluation and dissection of perigastric and nonperigastric fresh adipose tissue, according to the JGCA Protocol [2]. First, the different perigastric LN stations are isolated from the en bloc resection of stomach, omentum and lesser and greater curve adipose tissue, taking care not to leave any fat on the stomach wall (Figure 1A,B). In this phase, the surgeon indicated to the pathologist the blood vessels course and branching to identify the different LN stations. Following this, the surgeon detached by a scissor the adipose tissue of left gastric artery (station no. 7), hepatic pedicle (station no. 12), common hepatic artery (station no. 8), proximal splenic artery (station no. 11p) and splenic hilus (station no. 10), and coeliac axis (station no 9) by separating blood vessels, nerves, and LNs (Figure 1C). After, the perigastric and nonperigastric stations were isolated, the pathologist carefully examined them by a visual and a palpation phase and also by means of scraping off and dissecting the fat tissue by a scalpel. The joint collaboration with the surgeon allowed pathologist to focus along the vessel tiers where LNs are more abundant and easily identifiable by their peculiar morphology, color and consistency. Each LN was picked up and mapped one-by-one (Figure 1D). Finally, the surgical specimens were sent out in different boxes to the pathology lab for the standard grossing procedure.

As a second step, in the retrospective phase of the study, we collected the last 50 patients who had undergone a subtotal (either distal or proximal)/total gastrectomy plus D1 or D2 lymphadenectomy (depending on clinical conditions and tumor staging) before the beginning of the SARS-CoV-2 pandemic. The operative procedures in the control group were performed by the same surgeons but the JGCA protocol was applied only in 10 patients. The surgical specimens were represented by en bloc resection of stomach, omentum and lesser and greater curve adipose tissue, plus soft tissues surrounding common hepatic artery (station no. 8), celiac trunk (station no. 9), splenic hilus (station no 10), and splenic artery (station no. 11) in the D2 lymphadenectomy. The samples were immediately fixed and sent out to the pathology lab for examination. LNs were harvested by two different pathologists dedicated to gastrointestinal pathology following the classic manual method in the GR (not in the OR) on FF specimens (not on fresh tissue).

We analyzed the two groups in terms of number and maximum diameter of LNs dissected, as well as LNR. Since the number of LNs differs upon the site of dissection, LNs were grouped along the lymphatic compartment and the number of LNs belonging to each compartment were matched, if possible. Moreover, we compared the postoperative data to assess if an extended lymphadenectomy might affect the recovery and the short-term outcome. The following parameters were considered: need of and time spent in intensive care unit, postoperative complications according to Clavien–Dindo classification, length of hospitalization, 30-day mortality, OS.

Finally, we correlated the staff workload (including the time required for mapping) and the technical costs. In the prospective group, time for mapping was determined based on the time needed in OR and GR for harvesting and picking up all LNs. In the retrospective group, we considered the standard average time usually spent by pathologists for LNs sampling (i.e., 30 min).

For both groups, representative specimens of tumor, omentum and normal gastric tissue as well as of resection margins were performed, and followed standard and automatic procedures for processing and pathological examination.

After surgery, all the patients were discussed by a multidisciplinary team to define the postoperative approach according to the following guidelines [22]. The evaluation of the nutritional status was managed by specialized nutritionists. No patients were lost to follow-up.

### 2.2. Sample Size

We predicted a compliance rate of 67% would be achieved in this study similar to what was reported in previous studies examining conventional D2 LN dissection. The sample size was based on the alpha error at 0.05 and a power of 90%. As we set the equivalence difference to be 30% between the conventional and the proposed model, based on our pivotal communication [20], the total sample size required was calculated to be 43 patients for each group according to the formula. When we added 10% to mitigate expected follow-up loss, the total sample size was calculated to be 47 patients for each group.

### 2.3. Statistics

Statistical analysis was carried out by using commercially available statistical software (SPSS 24.0 for Windows SPSS Inc., Chicago, IL, USA) to calculate the association of epidemiologic and clinicopathological characteristics between the two groups. Chi-squared and Fisher’s exact test were performed for descriptive statistics. Quantitative variables were expressed as frequency count, minimum, maximum, mean, and standard deviation, while qualitative variables as frequency count and percentage. The Mann–Whitney-U test was used to compare continuous variables not normally distributed. All *p* values were two-sided with *p* < 0.05 considered statistically significant.

The following variables were included in the univariable analysis: gender (male vs. female), postoperative complications (yes vs. no), intensive care unit hospitalization (yes/no), days in care unit, length of hospitalization, 30-day mortality (yes/no). Survival function estimation and comparison between the two groups were performed using Kaplan–Meier estimates and log-rank test, respectively.

## 3. Results

The study group included 29 males (58%) and 21 females (42%), the mean age at diagnosis was 77 years (reference range: 65−87 years); six patients underwent urgency surgery for occlusive or bleeding disease; 15 patients were subjected to neoadjuvant chemotherapy following the FLOT (Fluorouracil, Leucovorin, Oxaliplatin and doceTaxel) protocol [22]. R0 resection was achieved in all cases. No adjacent organs (including pancreas, gallbladder and liver) were removed, except for two splenectomies for surgical trauma. The majority of tumors (*n* = 28, 56%) were located in the antral region, and were represented by low-grade (*n* = 19, 61%) [21], tubular poorly differentiated adenocarcinoma (*n* = 11, 22%) [21], intestinal type (*n* = 19, 38%) according to Lauren [23], poorly 1 (por1) (*n* = 11, 22%) following JGCA classification [24]. Interestingly, 16 poorly cohesive carcinoma (6 with signet ring phenotype and 10 belonging to other subtypes) were identified in our series. Four patients were diagnosed with early gastric cancer. Most patients suffered from an advanced disease presenting as pT4a (*n* = 25, 50%), with multiple nodal involvement and 10 metastatic cases. The most frequent stage according to the TNM VIII [6] and the AJCC 2017 [7] was IV (Table 1). These findings highlight the impact of the COVID-19 pandemic on cancer care, which has resulted in decreased early diagnosis and delayed treatment delivery.

**Figure 2:**
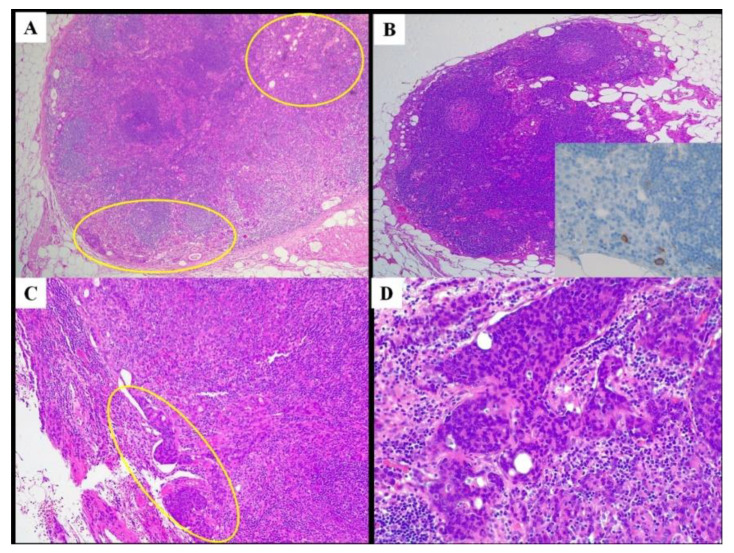
Metastatic lymph nodes. (**A**): one of the smallest metastatic lymph nodes (maximum diameter 1 mm), showing diffuse involvement (encircled). (**B**): isolated tumor cells found in a 1.4 mm-sized lymph node. (**B**, inset): immunohistochemistry highlighting neoplastic cells. (**C**): central lymph node metastasis (encircled). (**D**): skip metastasis in station number 7 by a patient with a lower antral tumor. A-D, hematoxylin and eosin stain; B, inset 8/18 cytokeratin stain. Original magnification: (**A**,**B**): 5×; (**C**): 10×; (**D**): 20×; (**B**), inset: 40×.

An average of 79 LNs were collected, with 77 LNs in subtotal and 83 LNs in total gastrectomy, respectively (Table 2). The compliance rate (i.e., cases when there was no more than one missing LN station during D2 LN dissection) was 78%. The maximum diameter ranged between 0.7 and 21 mm. The mean number of positive LNs was 7, showing a size between 0.7 and 12 mm (Figure 2A). We also identified very small metastases, the smallest one measuring 100 microns in maximum diameter, and we even isolated tumor cells (Figure 2B). Eight patients showed neoplastic involvement of CnLN (Figure 2C) and 11 patients showed skip metastases (Figure 2D).

The control group included 25 males and 25 females, the mean age at diagnosis was 74 years old (range: 37−92 years); 4 patients underwent urgency surgery for occlusive or bleeding disease; 5 patients were subjected to neoadjuvant chemotherapy according to the FLOT regimen. The majority of tumors (*n* = 30, 60%) were located in antral region, and were represented by low-grade (*n* = 19, 61%) [15], tubular moderately differentiated adenocarcinoma (*n* = 14, 28%) [21], intestinal type (*n* = 18, 36%) according to Lauren [23], tubular 2 (tub2) (*n* = 14, 28%) following JGCA classification [24]. Six patients suffered from early gastric cancer. Most of patients had a pT3 tumor (*n* = 30, 60%), with only 3 metastatic cases. The most frequent stage according to the TNM VIII [6] and the AJCC 2017 [7] was IIIA (*n* = 10, 20%) (Table 3).

The mean number of collected LNs was 29 (31 in total gastrectomy and 19 in subtotal gastrectomy), with a compliance rate of 54%. Maximum diameter ranged between 5 and 22 mm. An average number of 7 positive LNs were identified with micrometastasis and isolated tumor cells in 2 and 1 patients, respectively. Three patients who followed the JGCA protocol showed CnLN involvement, and one had skip metastasis (Table 4).

Our approach significantly increased the compliance rate for D2-lymphadenectomy (*p* = 0.05); accordingly, the mean number of LNs identified by the surgeon/pathologist team in OR was greater than the standard procedure of dissection carried out by the pathologist alone, with *p* = 0.001. Moreover, the smallest LNs were identified and harvested (*p* = 0.005), allowing for recognition of micrometastasis and isolated tumor cells. Interestingly, there was also a statistically significant difference in the number of LNs detected in the two groups based on a comparison of the lymphatic compartments (*p* = 0.05).

No intraoperative mortality was registered in the two groups. The overall postoperative complication rate requiring a second operation was not significantly different between the two groups (*n* = 5, 10% in the study group vs *n* = 6, 12% in the control one; *p* = 0.3). In the study group, complications were represented by bleeding (*n* = 3, 6%) and duodenal stump leakage (*n* = 2, 4%); whereas in the control group, 3 bleedings, 1 volvolus, 1 duodenal stump leak and 1 abdominal abscess occurred. Univariate analysis confirmed that age (*p* = 0.7), gender (*p* = 0.2), type of gastrectomy (*p* = 0.6), site of tumor (*p* = 0.5), histology (*p* = 0.3) and number of retrieved LNs (*p* = 0.4) are not related to morbidity. Complications mainly affected patients treated under emergency regimen (*p* = 0.001). Nine patients in the study group and 6 patients in the control group needed intensive care with at least one postoperative day (*p* = 0.3). Median hospital stay was 9 days in the study group and 11 days in the control one (*p* 0 0.5). The 30-day postoperative mortality was 8% (*n* = 4) in the study group and 14% (*n* = 7) in the control one (*p* = 0.4). Causes of death were: acute myocardial infarction (*n* = 1), disease progression (*n* = 1) following peritoneal carcinosis, uncontrolled bleeding (*n* = 2) despite embolization. In the control group, 1 patient diedfor acute pulmonary embolism, 1 due to intestinal volvolus leading to intestinal obstruction, 2 following acute myocardial infarction, and 3 for uncontrolled bleeding despite additional treatment. Univariate analysis showed that high 30-day mortality was related to age >75 years (*p* = 0.04), emergency regimen (*p* = 0.01), advanced stage (*p* = 0.02) and higher LNR (*p* = 0.02). These findings again support the feasibility of D2 lymphadenectomy, as overlap those reported for D1 lymphadenectomy [8].

After surgery, 22 out of 50 patients in the study group and 16 out of 50 in the control one underwent adjuvant chemotherapy according to the updated guidelines [22].

**Figure 3:**
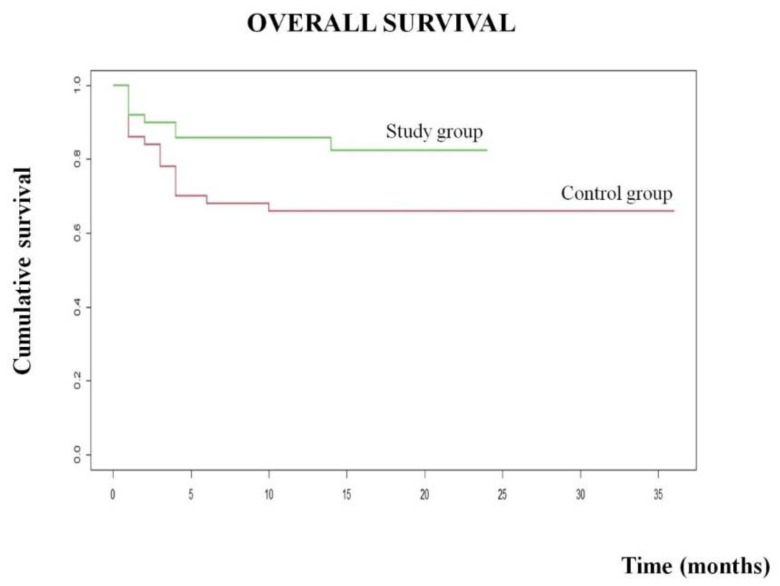
Overall survival of study group and control group.

In the study group, the OS rate was 85%, with a mean survival of 15 months in a total follow-up period of 24 months. Eight patients died, of these, 5for disease progression. In the control group, the OS rate was 66% with a mean survival of 25 months in a total follow-up period of 36 months. Ten out of 17 patients died following disease progression. In the univariate analysis, a significant mortality rate was related to age > 75 years (*p* = 0.02), urgency surgery (*p* = 0.01), advanced stage (*p* = 0.001), LN metastasis (*p* = 0.002), multiple LNs and CnLN involvement (*p* = 0.01 and *p* = 0.04) respectively), and skip metastasis (*p* = 0.03). Although no long-period follow-up is available to date to draw definite conslusions, this finding shows a better outcome in the study group patients (*p* = 0.03) (Figure 3), again confirming how a higher number of examined LNs is of paramount importance for a precise staging and a more tailored approach, which finally result in a better outcome.

As far as the feasibility of our approach is concerned, the proposed protocol by increasing the number of collected LNs obviously affects the technician and medical workload as well as the technical cost. Staff cost was not affected, as in Italy it is fixed. The time for sampling was shorter in the control than in the study group (30 vs. 45 min). Moreover, we observed an average increase of 20 blocks and 30 hematoxylin and eosin (H&E) slides per case to be prepared and examined. No streghthening in timing was detected for block processing, as in our lab all the procedures are fully automatized. However, technicians spent 40 additional minutes to section a case, and pathologist 15 additional minutes to examine the slides. In terms of technical costs, since EUR 3 is needed to produce an additional H&E slide, we observed an increase of 60 EUR per patient.

## 4. Discussion

LN stage is absolutely one of the leading prognostic factors influencing the OS after curative resection of GC [25]. According to the published guidelines, histopathological examination of at least 16 regional LNs is required to accurately assign the N category [2,6,7,22]. However, a number of studies, including clinical trials, has clearly demonstrated that within the same TNM stage, the greater the number of LN examined, the better is the prognosis [26]. A larger number of sampled LNs is directly related to a higher 5-year OS rate by also removing micrometastasis [5]. Inadequate or incomplete LNs dissection is implicated in understaging of patients [8,9,10] and higher probability of recurrence rate and metastases [5,13].

Gastrectomy and LNs harvesting and sampling are strictly operator-dependent. Although surgical procedures had been standardized, differences in the applied technique and in surgeon skills remain and may impact LNs removal. Moreover, in contrast to Eastern countries (i.e., China and Japan), D1-lymphadenectomy is still very common in Western countries, mainly because of lower rate of proximal stomach involvement and poorly cohesive histology [13]. The palpation approach, commonly used by pathologists in grossing procedures, is probably the most practiced method worldwide [27]. Usually, pathologist’s vigilance and insight are easily stimulated in cases of large tumors, while they are blunted for small ones [28,29,30,31]. Seeking to “find the expected” (i.e., metastatic LNs in large tumors) probably forces a more thorough analysis of the specimen [31]. On the other hand, the “search for the unlikely”, namely, metastatic nodes in early cancers, can subconsciously reduce the intensity of the effort to find scattered and often small LNs [31]. Accordingly, pathologist’s expertise is fundamental because the more experience one has, more accurate the evaluation will be. However, in skilled hands, 2−3 mm LNs could also be missed [27]. Various methods have been proposed to increase the efficiency of LNs harvesting, such as fat clearing, methylene blue staining, fat stretching, carbon nanoparticles, intraoperative radiation techniques with gamma probe, and a dedicated pathology assistant [27,28,29,30,31,32,33]. Although some of the aforementioned techniques appeared more efficient than others, there are insufficient data to assess whether a certain method can certainly improve LN count. The approach we proposed dramatically affected the number of detected LNs. In the study group, we removed a mean of 79 LNs against 29 in the control one. More interestingly, by comparing the study group patients with patients following the JGCA protocol in the control group, the difference in the number of harvested LNs was again statistically significative (79 vs. 31, *p* = 0.005), also when grouped by stations. “On site” evaluation of fresh tissue by the pathologist and surgeon allows for easier identification and grouping of the complex three-dimensional and multidirectional lymphatic system involved in GC [29,34,35]. In fact, there are things that only the surgeon knows, but similarly, there are features that only the pathologist recognizes. The surgeon precisely identifies the blood vessels’ course and branching and easily isolates the LN vascular bundles, thus facilitating the harvesting. On the other hand, the pathologist is skilled in distinguishing LNs by visualization (peculiar color and morphology) and palpation (firm consistency). Their joint cooperation could act as an extra motif for a more careful examination of the surgical sample. Interestingly, during the study period, we documented a progressive improvement in the respective skills, owing to the accumulation of cases.

Thanks to the technique we proposed, we picked up the smallest LNs and identified 2 SLM, 5 micrometastasis and two patients with isolated tumor cells [36], thus correctly staging all the patients undergoing the study. Moreover, we were able to provide additional parameters known to be helpful in prognostic stratification and optimal treatment planning, such as LNR, multiple LNs and CnLN involvement, and skip metastasis [37,38]. We found that patients with higher LNR, multiple LNs and CnLN involvement, and skip metastasis, experienced disease progression with shorter OS and higher mortality rate. Therefore, our study aids the concept that not only the N stage and the LNR, but also the anatomic extent of the positive LNs is strongly correlated with survival. The site of metastatic nodes marks regional spread of the disease and portents increased risk of recurrence and poorer prognosis [5]. Thus, the rationale of using the JGCA protocol [2] to provide a precise anatomical identification of the nodal stations, should be supported. Lastly, we confirmed the utility of D2 lymphadenectomy, as morbidity and mortality of our patients overlapped those reported for D1 lymphadenectomy. Interestingly, we found a better survival in the study group patients (85% vs. 66%; *p* = 0.03). This finding might be explained by the higher number of LNs removed, that not only reduced the mechanism of metastasis but also allowed for a precise staging and therapy [39].

The major issues we identified were the need for at least two dedicated pathologists who, in turn, should be present in the OR, and the increase in medical and technician workload and in technical costs. However, if considering the number and dimension of harvested LNs, the positive LNs identified and the better OS in the study group, we would not regard these tools as a drawback.

This study is not a randomized clinical trial and obviously suffers from some clear limitations, such as the low number of patients, the short-term follow-up, and the partial retrospective nature. Moreover, the advanced stage of patients belonging to the study group may, at least in part, accounts for the very high number of detected LNs if compared to the already published studies. However, our findings are statistically significant and strongly support the feasibility of the proposed technique, pending precise organization, skilled personnel and moderate-to-high volume centers. It is niteworthy that as we observed progressive improvement in medical skills during the study, we are aware that such an approach is also applicable in non-high-volume centers after a learning curve period for both the surgeon and pathologist.

## 5. Conclusions

It is fair to reiterate that suboptimal LN harvesting contributes to stage migration, inadequate treatment planning and poor outcome [40,41,42,43]. However, the hot question of how accomplish complete LNs removing still remains, as all the procedures proposed and applied so far have often been disappointing. Starting from the belief that the team approach is absolutely essential in improving the quality of care, we experienced a joint collaboration between surgeons and pathologists in OR that significantly increased the number of harvested LNs. By taking into account not only the TNM stage but also the extent of LN dissection, the LNR, and the topographical distribution of metastatic LNs, our approach improved the accuracy of patient management, finally leading to a higher survival rate.

We are aware that we cannot draw definite conclusions on the impact of this technique on oncological outcome; however, our findings support the notion that D2 lymphadenectomy is safe and achieves a better survival.

The technique we proposed seems to also be feasible in non-high-volume centers after accurate training of dedicated personnel; therefore, our study could serve as a proof-of-concept for a larger, multicentric collaboration.

## Figures and Tables

**Table 1:** Clinicopathological features, morbidity and mortality of study group patients.

N	G	A	SR	U	S	H	L	JGCA	GR	T	N	M	ST	P-O	M	OS
1	M	79	ST (D)	0	A	Tubular, poorly differentiated	IND	poorly 1	H	T3	N0	M0	IIA	0	1	20
2	M	87	ST (D)	0	AP	Poorly cohesive, NOS	D	poorly 2	H	T4a	N3b	M0	IIIC	0	0 (DP)	4
3	M	81	ST	0	C	Tubular, moderately differentiated	I	tubular 2	L	T2	N0	M0	II	0	1	24
4	F	65	TT	1 (O)	A	SRC	D	SRC	n.r.	yT4a	yN3a	yM1	IV	1 (L)	1	20
5	F	82	ST (D)	0	AP	Mixed	M	tubular 2 > poorly 2	n.r.	T4a	N3b	M0	IIIB	0	0 (AMI)	1
6	F	79	ST (D)	0	A	Tubular, well-differentated	I	tubular 1	L	T1b	N0	M0	IA	0	1	16
7	F	76	TT	0	Ang	Poorly cohesive, NOS	D	poorly 2	n.r.	yT4a	yN3b	yM1	IV	0	1	6
8	M	76	ST (D)	0	A	Tubular, moderately differentiated	I	tubular 2	L	yT3	yN2	yM0	IIIA	0	1	7
9	M	68	ST (D)	0	A	Poorly cohesive, NOS	D	poorly 2	n.r.	T4a	N3b	M0	IIIC	0	0 (DP)	1
10	M	72	ST (D)	0	CA	SRC	D	SRC	n.r.	yT0	yN0	yM0	IB	0	1	18
11	M	77	ST (D)	1 (B)	P	Tubular, moderately differentiated	I	tubular 2	L	T4a	N2	M0	IIIA	1 (B)	1	9
12	F	85	ST (D)	0	A	Tubular, poorly differentiated	IND	poorly 1	H	T4a	N3b	M1	IV	0	0 (DP)	4
13	F	77	ST (D)	0	A	Tubular, well-differentiated	I	tubular 1	L	T1a	N0	M0	IA	0	1	22
14	M	77	TT	0	C	Poorly cohesive, NOS	D	poorly 2	n.r.	yT4b	yN2	yM1	IV	0	1	10
15	M	80	ST (D)	1 (B)	A	Tubular, poorly differentiated	IND	poorly 1	H	T4b	N3b	M1	IVB	1 (B)	0 (B)	1
16	F	84	ST (D)	0	A	Mixed	M	tubular, poorly differentiated > SRC	n.r.	T4a	N3b	M0	IIIC	0	1	11
17	F	76	ST (D)	1 (B)	A	Tubular, poorly differentiated	IND	poorly 1	H	yT2	yN0	yM0	IB	1 (L)	1	14
18	M	70	ST (D)	0	A	Tubular, moderately differentiated	I	tubular 2	L	T4a	N3b	M0	IIIC	0	1	4
19	M	76	TT	0	FC	SRC	D	SRC	n.r.	T4a	N3a	M1	IV	0	1	7
20	F	72	ST (D)	1 (O)	CA	Tubular, poorly differentiated	IND	poorly 1	H	T4a	N2	M0	IIIA	1 (B)	0 (B)	1
21	M	81	TT	0	CA	Mixed	M	tubular, moderately differentiated > poorly cohesive, NOS	n.r.	T4a	N3a	M1	IV	0	1	13
22	M	76	ST (D)	0	A	Tubular, well-differentiated	I	tubular 1	L	T1a	NX	M0	IA	0	1	23
23	M	76	ST (D)	0	A	Tubular, moderately differentiated	I	tubular 2	L	T1b	N0	M0	IA	0	1	22
24	M	80	ST (D)	0	P	SRC	D	SRC	n.r.	T4a	N0	M0	IIB	0	1	16
25	F	86	TT	0	FC	Papillary	I	papillary	L	T3	N0	M0	IIA	0	1	24
26	M	78	ST (D)	0	A	Tubular, poorly differentiated	IND	poorly 1	H	T4a	N3a	M0	IIIC	0	1	13
27	M	74	TT	0	CA	Papillary	I	papillary	L	T3	N0	M0	IIA	0	1	23
28	F	81	TT	0	CA	SRC	D	SRC	n.r.	T4a	N3b	M1	IV	0	0 (DP)	14
29	F	84	ST (D)	0	A	Tubular, poorly differentiated	IND	poorly 1	H	T2	N3a	M0	IIIA	0	1	6
30	M	86	ST (D)	0	A	Tubular, moderately differentiated	I	tubular 2	L	T4a	N1	M0	IIIA	0	0 (DP)	2
31	M	72	ST (D)	0	A	Tubular, moderately differentiated	I	tubular 2	L	T2	N0	M0	IB	0	1	23
32	M	84	TT	0	A	Poorly cohesive, NOS	IND	poorly 2	n.r.	yT4a	yN3a	yM0	IIIB	0	1	9
33	M	86	ST (D)	0	A	Tubular, well-differentiated	I	tubular 1	L	T3	N0	M0	IIA	0	1	24
34	F	69	TT	0	A	Poorly cohesive, NOS	IND	poorly 2	n.r.	yT4a	yN3b	yM0	IIIC	0	1	4
35	F	84	TT	0	C	Tubular, poorly differentiated	IND	poorly 1	H	T3	N1	M0	IIB	0	1	14
3	F	76	ST (D)	0	C	Tubular, moderately differentiated	I	tubular 2	L	T3	N0	M0	IIA	0	1	24
37	M	72	TT	0	A	Mixed	M	tubular 2 > poorly 2	n.r.	T3	N0	M0	IIA	0	1	20
38	M	78	ST (D)	0	A	Tubular, well-differentiated	I	tubular 1	L	T2	N0	M0	IB	0	1	24
39	M	78	ST (P)	0	C	Tubular, well-differentiated	I	tubular 1	L	T2	N0	M0	IB	0	1	24
40	F	86	TT	0	A	Poorly cohesive, NOS	D	poorly 2	n.r.	yT4a	yN2	yM0	IIIA	0	1	11
41	M	87	TT	0	C	Tubular, poorly differentiated	IND	poorly 1	H	yT4a	yN0	yM0	IIB	0	1	16
42	F	76	TT	0	A	Poorly cohesive, NOS	D	poorly 2	n.r.	yT4a	yN1	yM0	IIIA	0	1	9
43	F	71	TT	0	C	SRC	D	SRC	n.r.	yT4a	yN2	yM0	IIIA	0	1	7
44	M	77	ST (D)	0	A	Tubular, moderately differentiated	I	tubular 2	L	T2	N0	M0	IB	0	1	24
45	M	71	ST (D)	1 (B)	P	Tubular, well-differentiated	I	tubular 1	L	T3	N0	M0	IIA	0	1	23
46	F	80	TT	0	CA	Poorly cohesive, NOS	D	poorly 2	n.r.	yT4a	N0	yM1	IV	0	1	6
47	F	84	ST (D)	0	A	Tubular, moderately differentiated	I	tubular 2	L	T3	N0	M0	IIA	0	1	18
48	F	82	ST (D)	0	A	Tubular, poorly differentiated	IND	poorly 1	H	T3	N1	M0	IIB	0	1	12
49	M	75	TT	0	CA	Poorly cohesive, NOS	D	poorly 2	n.r.	yT4a	yN2	yM1	IV	0	1	7
50	M	78	ST (D)	0	A	Tubular, poorly differentiated	IND	poorly 1	H	yT4a	yN0	yM0	IIB	0	1	11

G: gender; A: age; SR: surgery; U: urgency; S: site; H: histotype (WHO 2019); L: Lauren; JGCA: Japanese Gastric Cancer Association; GR: grading (WHO 2019); ST: stage (TNM VIII/AJCC 2017); PO: postoperative complications; M: mortality; OS: overall survival; 0: absence; 1: presence; M: male; F: female; ST: subtotal; D: distal; P: proximal; TT: total; F: fundus; C: corpus; ANG: angulus; A: antrum; P: pylorus; I: intestinal; D: diffuse; IND: indeterminate; M: mixed; SRC: signet-ring cell; L: low grade; H: high grade; DP: disease progression; AMI: acute myocardial infarction; B: bleeding; L: duodenal stump leak; n.r.: not required.

**Table 2:** Lymph nodes dissection according to the J.G.C.A. protocol in the study group.

N	St 1	St 2	St 3	St 4sa	St 4sb	St 4d	St 5	St 6	St 7	St 8	St 9	St 10	St 11	St 12	LNR
1	0	n.p.	0/18	n.p.	0/5	0/10	0/11	0/15	0/8	0/17	0	n.p.	0	0	0/84
2	0	n.p.	7/15	n.p.	0/2	0/1	3/6	4/10	4/26	0/2	0	n.p.	1/1	0	19/63
3	0/4	0/13	0/3	0/10	0/5	n.p.	n.p.	n.p.	0/1	0	0	n.p.	0	n.p.	0/36
4	0/6	0/2	5/13	0/1	0/5	4/5	0/1	0/10	0/9	0/2	0/1	n.p.	0	0	9/55
5	0/3	n.p.	3/20	n.p.	0/16	2/9	0/2	9/26	0/14	0/10	0/3	n.p.	0/2	0/2	14/107
6	0/2	n.p.	0/11	n.p.	0/9	0/12	0/3	0/6	0/2	0/4	0/2	n.p.	n.p.	n.p.	0/51
7	3/10	0/2	0/10	0/10	1/7	10/12	3/10	0/1	3/12	1/13	0/2	n.p.	0	0/1	21/90
8	0/2	n.p.	0/13	n.p.	3/13	0/43	0/8	0/1	0/18	0/2	0/1	n.p.	0/3	0	3/104
9	15/20	n.p.	1/1	n.p.	0/9	4/21	0/1	7/13	1/1	18/21	0/3	n.p.	0	0/2	46/92
10	0/7	n.p.	0/15	n.p.	0/11	0/14	0/2	0/16	0/3	0/11	0/3	n.p.	0/1	0/2	0/85
11	0	n.p.	0/10	n.p.	0/15	0/11	0/2	3/19	0/6	0/2	0/1	n.p.	0	0	3/66
12	10/25	n.p.	8/9	n.p.	0/5	8/15	2/5	8/18	0/1	3/3	1/1	n.p.	0	1/1	40/83
13	0	n.p.	0/2	n.p.	0/5	0/5	0/2	0/12	0/2	0/3	n.p.	n.p.	n.p.	n.p.	0/31
14	0/12	0	3/23	0/1	1/32	0/5	0/1	0/10	0/15	0/5	0/2	0/2	0/8	0/4	4/120
15	0/1	n.p.	23/23	n.p.	3/3	2/3	3/3	19/19	5/8	6/6	0/1	n.p.	0	0	61/67
16	0/21	n.p.	5/7	n.p.	3/17	0/18	3/3	0/6	3/3	0/3	0/1	n.p.	0	0/1	14/80
17	0/6	n.p.	0/26	n.p.	0/2	0/19	0/8	0/20	0/13	0/10	0/19	n.p.	0/2	0/1	0/126
18	4/17	n.p.	0/1	n.p.	0/31	0/9	2/4	15/34	1/3	0/5	0/6	n.p.	1/9	0/2	23/121
19	2/7	0/6	7/18	0/3	0/1	0/8	0/3	0/3	0/1	0	0	n.p.	0	0	9/50
20	0/3	n.p.	2/15	n.p.	0/3	0/17	0/17	1/48	0/13	0/8	0/3	n.p.	0/11	0/1	3/145
21	0/3	1/3	1/6	0/4	0/4	4/6	1/5	0/2	0/2	0/2	0/1	n.p.	0	0/1	7/39
22	0	n.p.	0/5	n.p.	0/8	0/6	0/2	0/10	0/1	n.p.	n.p.	n.p.	n.p.	n.p.	0/32
23	0	n.p.	0	n.p.	0/5	0/4	0/2	0/13	0/9	n.p.	n.p.	n.p.	n.p.	n.p.	0/33
24	0/5	n.p.	0/2	n.p.	0/26	0/4	0/1	0/12	0/14	0/3	0/3	0/4	0	0/1	0/75
25	0/4	0/4	0/7	0/4	0/4	n.p.	0	n.p.	0/2	0/2	0/2	n.p.	0/2	0	0/31
26	0	n.p.	0	n.p.	0/5	0/15	7/13	0/16	2/9	1/1	0/1	n.p.	0	0	10/60
27	0	0/1	0/8	0/2	0/2	0/1	0/31	0/1	0/1	0/4	0	n.p.	0	0	0/45
28	1/1	2/2	3/4	3/3	0/1	3/6	1/1	2/8	1/1	1/2	1/1	n.p.	1/1	0	19/31
29	2/6	n.p.	2/11	n.p.	0/7	0/15	0/5	0/10	7/14	0/3	0/2	n.p.	0	0/1	11/74
30	0/1	n.p.	0/8	n.p.	0/3	0/1	0/2	1/3	1/3	1/3	1/2	n.p.	1/3	0/2	5/31
31	0/1	n.p.	0/1	n.p.	0/1	0/6	0/5	0/8	0/1	0/7	0/1	n.p.	0	0/2	0/33
32	0/6	0/6	0/4	0/16	2/18	2/20	0/18	4/28	0/6	0/2	0/14	n.p.	0/1	0/1	8/130
33	0/2	n.p.	0/2	n.p.	0/16	0/14	0/18	0/20	0/4	0/1	0/1	n.p.	0/1	0/1	0/80
34	0/2	0/3	0/5	0/7	0/10	6/24	2/26	10/28	0/3	0/4	0/4	n.p.	0/2	0/2	18/120
35	0/4	0/2	2/16	0/10	0/9	0/12	0/16	0/15	0/4	0/1	0/1	n.p.	0/1	0/1	2/92
36	0/6	n.p.	0/8	n.p.	0/13	0/12	0/15	0/13	0/4	0/2	0/1	n.p.	0/1	0/1	0/86
37	0/4	0/2	0/2	0/8	0/12	0/16	0/18	0/7	0/3	0/2	0/1	n.p.	0/1	0/1	0/87
38	0/2	n.p.	0/2	n.p.	0/19	0/21	0/21	0/16	0/1	0/1	0/1	n.p.	0	0	0/84
39	0/3	0/5	0/16	0/20	0/26	0/5	n.p.	n.p.	n.p	0/1	0/1	n.p.	0/1	0/1	0/79
40	0/6	0/6	0/4	0/16	0/20	2/24	0/21	3/32	0/4	0/2	0/2	n.p.	0/1	0/2	5/140
41	0/2	0/2	0/14	0/12	0/7	0/11	0/13	0/14	0/3	0/2	0/1	n.p.	0/1	0	0/82
42	0/2	0/2	0/2	0/1	0/4	0/18	0/24	1/26	0/2	0/2	0/1	n.p.	0/1	0/1	1/86
43	0/6	0/6	2/24	2/35	0/20	0/12	0/6	0/10	0/6	0/2	0/1	n.p.	0/1	0/1	4/130
44	0/6	n.p.	0/8	n.p.	0/10	0/20	0/11	0/26	0/1	0/1	0	n.p.	0/2	0/1	0/88
45	0/4	n.p.	0/8	n.p.	0/14	0/18	0/9	0/25	0/2	0/2	0/2	n.p.	0	0/2	0/86
46	0/4	0/4	0/5	0/6	0/16	0/17	0/9	0/22	0/3	0/1	0/1	n.p.	0	0/1	0/89
47	0/2	n.p.	0/4	n.p.	0/22	0/20	0/10	0/24	0/3	0/2	0/1	n.p.	0	0/1	0/89
48	0/2	n.p.	0/1	n.p.	0/24	0/26	0/12	2/28	0/2	0/1	0	n.p.	0/1	1	2/98
49	0/2	0/2	0/3	0/5	0/23	1/32	0/9	2/34	0/4	0/1	0	n.p.	0/2	0/2	3/119
50	0/2	n.p.	0/3	n.p.	0/16	0/14	0/18	0/20	0/4	0/1	0/1	n.p.	0/1	0/1	0/81

St: station number; n.p.: not performed; LNR: lymph node ratio.

**Table 3:** Clinicopathological features, morbidity and mortality of control group patients.

N	G	A	SR	U	S	H	L	JGCA	GR	T	N	M	ST	P-O	M	OS
1	M	80	ST (P)	0	FC	Tubular, moderately differentiated	I	tubular 2	L	T4a	N3a	M1	IV	0	0 (DP)	4
2	M	59	ST (D)	0	A	Mixed	M	tubular 2 > poorly 2	n.r.	T3	N2	M0	IIIA	0	1	20
3	F	77	ST (D)	0	A	Mixed	M	mucinous > poorly 2	n.r.	T3	N2	M0	IIIA	0	0 (DP)	10
4	F	75	ST (D)	0	A	Mixed	M	poorly 1 > poorly 2	n.r.	yT4a	yN3b	yM0	IIIC	0	1	16
5	F	57	ST (D)	0	A	Mixed	M	mucinous > poorly 2	n.r.	yT3	yN0	yM0	IIA	0	1	34
6	M	71	ST (D)	0	A	Tubular, well-differentiated	I	tubular 1	L	T1b	N0	M0	IA	0	1	30
7	F	78	TT	0	C	Tubular, poorly differentiated	IND	poorly 1	H	T4a	N3a	M0	IIIB	0	0 (DP)	3
8	M	83	TT	0	CA	Mixed	M	poorly 1 > micropapillary	n.r.	T3	N2	M0	IIIA	0	1	18
9	M	77	TT	1 (B)	C	Tubular, poorly differentiated	IND	poorly 1	H	T3	N3b	M0	IIIC	1 (B)	0 (B)	1
10	M	78	TT	1 (B)	ANG	Poorly cohesive, NOS	D	poorly 2	n.r.	T3	N3a	M0	IIIB	1 (B)	0 (B)	1
11	M	58	ST (D)	0	A	Tubular, moderately differentiated	I	tubular 2	L	T3	N0	M0	IIA	0	1	28
12	M	76	ST (D)	0	A	Tubular, moderately differentiated	I	tubular 2	L	T1a	N0	M0	IA	0	1	32
13	F	86	ST (D)	0	A	Tubular, moderately differentiated	I	tubular 2	L	T4a	N1	M0	IIIA	0	1	18
14	F	37	TT	0	CA	Mixed	M	poorly 1 > poorly 2	n.r.	T3	N3a	M0	IIIB	1 (V)	0 (DP)	6
15	F	81	ST (D)	1 (O)	AP	SRC	D	SRC	H	T4a	N3b	M0	IIIC	0	0 (V)	1
16	M	80	ST (P)	0	FC	Tubular, poorly differentiated	IND	poorly 1	H	T1b	N0	M0	IA	0	1	34
17	M	70	ST (D)	0	A	Mucinous	I	mucinous	L	T3	N2	M0	IIIA	0	1	24
18	F	67	TT	0	A	SRC	D	SRC	n.r.	yT4a	yN3a	yM1	IV	0	0 (DP)	4
19	M	83	ST (D)	0	A	Tubular, moderately differentiated	I	tubular 2	L	T3	N0	M0	IIA	0	1	36
20	F	80	TT	0	CA	Poorly cohesive, NOS	D	poorly 2	n.r.	T3	N2	M0	IIIA	0	1	18
21	M	82	TT	0	CA	Tubular, poorly differentiated	IND	poorly 1	H	T3	N1	M0	IIB	0	1	24
22	F	56	TT	0	A	Tubular, poorly differentiated	IND	poorly 1	H	T3	N1	M0	IIB	0	1	24
23	M	75	TT	0	A	Tubular, poorly differentiated	IND	poorly 1	H	T4a	N3b	M0	IIIC	0	0 (DP)	4
24	M	83	ST (D)	0	A	Papillary	I	papillary	L	T1a	N0	M0	IA	0	1	34
25	M	75	TT	0	A	Poorly cohesive, NOS	D	poorly 2	H	T4a	N2	M0	IIIA	0	1	18
26	M	83	ST (D)	0	A	Tubular, moderately differentiated	I	tubular 2	L	T3	N1	M0	IIB	1 (ABS)	1	28
27	M	68	TT	0	A	SRC	D	SRC	n.r.	T3	N3b	M0	IIIC	0	0 (DP)	3
28	M	74	ST (D)	0	A	Tubular, moderately differentiated	I	tubular 2	L	T1a	N0	M0	IA	0	1	32
29	M	77	TT	0	A	Mixed	M	poorly 1 > SRC	n.r.	T3	N3b	M1	IV	0	0 (DP)	2
30	F	81	TT	0	A	Tubular, poorly differentiated	IND	poorly 1	H	T3	N3a	M0	IIIB	1 (B)	1	18
31	F	59	ST (D)	1 (B)	A	Tubular, moderately differentiated	I	tubular 2	L	T3	N3a	M0	IIIB	0	0 (B)	1
32	F	72	ST (D)	0	A	Tubular, moderately differentiated	I	tubular 2	L	T3	N0	M0	IIA	0	1	30
33	F	82	ST (D)	0	C	Mucinous	D	mucinous	L	T3	N1	M0	IIB	0	1	18
34	F	78	ST (D)	0	A	Mucinous	D	mucinous	H	yT4a	yN3b	yM0	IIIC	0	0 (PE)	1
35	F	79	ST (D)	0	A	Mixed	M	poorly 1 > poorly 2	n.r.	T4a	N3a	M0	IIIB	0	1	18
36	M	74	ST (D)	0	P	Tubular, moderately differentiated	I	tubular 2	L	T3	N1	M0	IIB	0	1	26
37	M	78	ST (D)	0	C	Tubular, well-differentiated	I	tubular 1	L	T3	N0	M0	IIA	0	1	34
38	F	74	TT	0	ANG	Tubular, moderately differentiated	I	tubular 2	L	T3	N1	M0	IIB	0	1	24
39	F	82	ST (D)	0	A	Mixed	M	tubular 2 > mucinous	n.r.	T3	N3a	M0	IIIB	0	1	16
40	F	72	ST (D)	0	A	Mixed	M	poorly 1 > SRC	n.r	T2	N3b	M0	IIIB	0	0 (DP)	3
41	F	74	ST (D)	0	P	Tubular, poorly differentiated	IND	poorly 1	H	T3	N2	M0	IIIA	0	1	18
42	M	78	ST (P)	0	FC	Tubular, poorly differentiated	IND	poorly 1	H	T3	N0	M0	IIA	0	1	34
43	F	75	TT	0	A	Mixed	M	poorly 1 > poorly 2	n.r.	T4a	N3a	M0	IIIB	1 (L)	1	16
44	F	85	ST (D)	0	A	Tubular, moderately differentiated	I	tubular 2	L	T3	N3b	M0	IIIC	0	0 (AMI)	1
45	F	75	ST (D)	0	AP	Mixed	M	poorly 1 > poorly 2	n.r.	T3	N2	M0	IIIA	0	1	18
46	M	60	ST (D)	0	AP	Tubular, moderately differentiated	I	tubular 2	L	T3	N1	M0	IIB	0	1	36
47	F	84	ST (D)	0	A	Tubular, moderately differentiated	I	tubular 2	L	T1b	N1	M0	IB	0	1	36
48	M	72	TT	0	C	Mixed	M	poorly 1 > poorly 2	n.r.	T2	N1	M0	IIA	0	1	30
49	F	92	TT	0	A	SRC	D	SRC	n.r	yT4b	yN2	yM0	IIIB	0	0 (AMI)	1
50	M	60	TT	0	C	Poorly cohesive, NOS	D	poorly 2	n.r.	T3	N3b	M0	IIIC	0	0 (DP)	4

G: gender; A: age; SR: surgery; U: urgency; S: site; H: histotype (WHO 2019); L: Lauren; JGCA: Japanese Gastric Cancer Association; GR: grading (WHO 2019); ST: stage (TNM VIII/AJCC 2017); PO: postoperative complications; M: mortality; OS: overall survival; 0: absence; 1: presence; M: male; F: female; ST: subtotal; D: distal; P: proximal; TT: total; F: fundus; C: corpus; ANG: angulus; A: antrum; P: pylorus; I: intestinal; D: diffuse; IND: indeterminate; M: mixed; SRC: signet-ring cell; L: low grade; H: high grade; n.r.: not required; B: bleeding; V: volvolus; O: obstruction; DP: disease progression; PE: pulmonary embolism; AMI: acute myocardial infarction.

**Table 4:** Lymph nodes dissection in the control group.

N	LC	GC	Other	LNR
1	6/30	1/7		7/37
2	2/45	0/5	1/13	3/63
3	6/10	0/5	0/5	6/20
4	24/47	4/11	7/7	35/65
5	0/2	0/54		0/56
6	0/12	0/10		0/22
7	0/4	11/20	0/2	11/26
8	4/19	0/12		4/31
9	1/6	17/20		18/26
10	0/4	14/56		14/30
11	0/5	0/40		0/19
12	0/13	0/19		0/32
13	1/12	0/15	0/13	1/40
14	5/18	5/14	0/7	10/39
15	1/5	15/15	2/7	18/27
16	0/9	0/9		0/18
17	0/5	5/21		5/26
18	7/21	7/9	0/8	14/38
19	0/17	0/8		0/25
20	0/5	4/20	0/1	4/26
21	0/3	1/19	0/1	1/23
22	0/13	2/28	0/1	2/42
23	6/18	10/17		16/35
24	0/6	0/4		0/20
25	0/5	4/16	0/7	4/28
26	0/18	1/15	0/1	1/34
27	10/22	7/13	1/3	18/38
28	0/19	0/5		0/24
29	5/11	14/18	0/2	19/31
30	5/17	5/14		10/31
31	8/12	4/7	1/1	13/20
32	0/7	0/14	0/1	0/22
33	0/16	1/14	0/10	1/40
34	17/21	1/5	0/2	18/28
35	2/7	7/17	0/3	9/27
36	2/5	0/12		2/17
37	0/15	0/5	0/17	0/37
38	0/9	1/13	0/6	1/28
39	7/20	6/8	0/1	13/29
40	26/28	9/14		35/42
41	1/12	2/5		3/17
42	0/9	0/10	0/4	0/23
43	8/10	0/7	1/1	9/18
44	13/17	10/12		23/29
45	2/14	3/14		5/28
46	0/19	1/7	0/1	1/27
47	1/8	0/7		1/15
48	1/7	0/5	0/4	1/17
49	6/10	0/6		6/16
50	7/13	20/16		27/29

LC: lesser curve; GC: greater curve; LNR: lymph node ratio.

## Data Availability

All the information regarding the patients is available in the database of Pathology and Surgery Unit of Azienda Toscana Nord-Ovest.
